# The genetic epidemiology of idiopathic scoliosis

**DOI:** 10.1007/s00586-012-2389-6

**Published:** 2012-06-14

**Authors:** Kristen Fay Gorman, Cédric Julien, Alain Moreau

**Affiliations:** 1 Viscogliosi Laboratory in Molecular Genetics of Musculoskeletal Diseases, Sainte Justine University Hospital Research Center, Montreal, QC Canada; 2 Department of Biochemistry, Faculty of Medicine, University of Montreal, Montreal, QC Canada; 3 Department of Stomatology, Faculty of Dentistry, University of Montreal, Montreal, QC Canada

**Keywords:** Genetics, Genes, Epidemiology, Idiopathic scoliosis, Adolescent idiopathic scoliosis, Spinal curvatures

## Abstract

**Purpose:**

Idiopathic scoliosis is a complex developmental syndrome defined by an abnormal structural curvature of the spine. High treatment costs, chronic pain/discomfort, and the need for monitoring at-risk individuals contribute to the global healthcare burden of this musculoskeletal disease. Although many studies have endeavored to identify underlying genes, little progress has been made in understanding the etiopathogenesis. The objective of this comprehensive review was to summarize genetic associations/linkages with idiopathic scoliosis, as well as explore the strengths and weaknesses of each study, such that it may serve as a guide for the design and interpretation of future genetic studies in scoliosis.

**Methods:**

We searched PubMed and Human Genome Epidemiology (HuGE) Navigator using the search terms “gene and scoliosis”. Linkage or association studies published in English and available full-text were further analyzed as regards results, experimental design, and statistical approach.

**Results:**

We identified and analyzed 50 studies matching our criteria. These consisted of 34 candidate gene studies (6 linkage, 28 association) and 16 genome-wide studies [14 pedigree-based linkage, 2 genome-wide association studies (GWAS)]. Findings involved genes related to connective tissue structure, bone formation/metabolism, melatonin signaling pathways, puberty and growth, and axon guidance pathways. Variability in results between studies suggested ethnic and/or genetic heterogeneity.

**Conclusions:**

The major difficulty in idiopathic scoliosis research is phenotypic and genetic heterogeneity. Genetic research was overrepresented by underpowered studies. The use of biological endophenotypes, as well as restricted clinical definitions, may help to partition variation and increase the power of studies to detect or confirm an effect.

## Introduction

Idiopathic scoliosis (IS) is a complex developmental syndrome that constitutes the largest subgroup of human spinal curvatures [Online Mendelian Inheritance in Man (OMIM): 181800]. First described by Hippocrates in *On the Articulations* (Part 47), IS has been the subject of ongoing research, and yet its etiology remains enigmatic. IS is marked by phenotypic complexity (variations in curve morphology and magnitude, age of onset, rate of progression), and a prognosis ranging from increase in curve magnitude, to stabilization, or to resolution with growth. Genetic factors are known to play a role, as observed in twin studies and singleton multigenerational families [1]. A recent study of monozygotic and dizygotic twins from the Swedish twin registry estimated that overall genetic effects accounted for 38 % of the observed phenotypic variance, leaving the remaining 62 % to environmental influences [2]. Genetic complexity in IS is further inferred from inconsistent inheritance [3–6], discordance among monozygotic twins [7–9], and highly variable results from genetic studies.

The standard of care for scoliosis has not changed significantly in the past three decades, from initiating observation to bracing and to spinal fusion surgery as a last resort [10]. The healthcare costs of bracing, hospitalization, surgery, and chronic back pain are substantial. An understanding of the genetics underlying the disorder would help lead to earlier diagnosis, identification of at-risk individuals, and more effective preventive and/or therapeutic choices.

Genetic variants that can affect a person’s predisposition to spinal curvature and the propensity for progression to severe curvature are still unknown. Since 1992, over 60 studies have attempted to identify genes by either genome-wide or hypothesis-driven designs, using either pedigrees (linkage analysis) or unrelated case–control population samples (association studies). Of over 30 candidate genes tested, 18 unique loci have been identified, suggesting that IS may be caused by multiple genes segregating differently in various populations. The goal of this review was to evaluate the various genetic studies and amalgamate their results to provide new insights. As reviewing genetic studies in a complex syndrome such as IS requires an evaluation of study design, and not merely a reporting of the findings [11], this comprehensive review may also serve as a guide for the design and interpretation of future genetic studies in IS [12].

## Methods

We conducted a literature search of the PubMed database using the term “gene and scoliosis” to retrieve genetic studies in IS published between 1992 and 2011. Studies published in English and available as full-text were considered for further analysis if they referred to either association or linkage studies. The search was replicated using Human Genome Epidemiology (HuGE) Navigator, version 2.0 [13].

We evaluated the quality of the experimental design as follows: For association studies, we examined the number of individuals included (case and control groups), whether/how the phenotype was defined (female only, gender-matched, gender not defined; minimum curve magnitude considered; whether the phenotype was subcategorized; whether the phenotype was confirmed by physical examination, radiograph, or questionnaire), and the statistics employed (correction for multiple testing when appropriate; power analysis when appropriate). For linkage studies, we considered the number and size of families, how the phenotype was defined, the design of the study (parametric or nonparametric analysis), and the strength of the linkage.

## Results

**Fig. 1:**
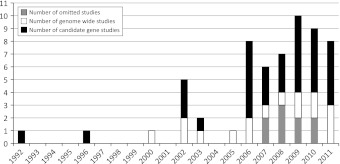
Survey of genetic studies in idiopathic scoliosis. Efforts to identify genes for idiopathic scoliosis have largely been hypothesis-driven candidate gene studies, the majority of which were association studies. Year-by-year results are shown. Most of the genome-wide studies were linkage studies. Genome-wide studies do not presume a hypothesis to locate associated or linked chromosomal regions, while candidate gene studies examine the effects of specific gene variants hypothetically involved in the disease. Candidate gene studies are shown in *black* (*n* = 34), genome-wide studies in *white* (*n* = 16); studies not analyzed in this review are shown in *gray* (*n* = 9)

We found 50 articles that matched our criteria. These consisted of 34 candidate gene studies (6 linkage studies and 28 association studies) as well as 16 genome-wide studies [14 pedigree-based linkage studies and 2 genome-wide association studies (GWAS)]. Nine other candidate gene studies were not considered for analysis because they did not satisfy inclusion criteria (were not published in English or available as full text articles). A yearly breakdown of all studies identified is shown in Fig. 1.

## Candidate gene studies

The selection of candidate genes for study can be made based on biological systems possibly playing a role in the etiopathogenesis of a disorder (from clinical evaluations or animal research), previous genetic studies showing an association (replication studies), or positional information gained from linkage studies (in combination with hypotheses). Between 1992 and 2006, many candidate gene studies for IS were family-based linkage studies [14–19]. Whereas family-based methods of hypothesis testing are more efficient at finding variants underlying rare conditions or rare subphenotypes of a common condition [20], they have largely been abandoned in the study of complex diseases due to their low power for detecting common variants [21, 22]. This may explain why, after 2006, case–control association studies constituted the bulk of candidate gene research.

For the included studies, we subdivided candidate genes by category reflecting their hypothetical functional involvement in IS: connective tissue structure, bone formation and bone metabolism, melatonin signaling pathway, and puberty and growth (Table 1).

**Table 1:** Candidate gene studies for idiopathic scoliosis

Gene	Number of inquiries	Number of positive associations [References]	Number of negative associations [References]
Connective tissue structure			
FBN1	1	0	1 [14]
ELN	1	0	1 [14]
COL1A1	1	0	1 [14]
COL1A2	2	0	2 [14, 15]
COL2A1	1	0	1 [14]
ACAN	2	0	2 [16, 18]
MATN1	3	2 [19, 22]	1 [23]
LOX1, LOX2, LOX3, LOX4, LOX5	1	0	1 [24]
TIMP2	1	1 [30]	0
MMP3	3	1 [31]	2 [32, 33]
DPP9	1	0	1 [37]
Bone formation/metabolism			
BMP4	1	0	1 [33]
LEP	1	0	1 [33]
CALM1	1	1 [45]	0
IL6	4	2 [31, 52]	1 [33]*
VDR	2	1 [53]	1 [54]
TNFRSF11B (OPG)	1	1 [55]	0
RANKL	1	0	1 [55]
RANK	1	0	1 [55]
Melatonin signaling pathway			
MTNR1A	3	0	3 [17, 59, 60]
MTNR1B	6	1 [62]	5 [23, 33, 60, 61, 63]
TPH1	3	1 [64]	2 [23, 60]
ASMT (HIOMT)	1	0	1 [60]
AANAT (SNAT)	2	0	2 [60, 64]
GPR50	1	0	1 [63]
Puberty and growth			
CYP17	1	0	1 [54]
ESR1 (alpha)	6	4 [45, 67–69]	2 [70, 71]
ESR2 (beta)	2	1 [73]	1 [71]
GPER (GPR30)	1	1 [74]	0
GHR	2	0	3 [76, 78]
IGF1	3	1 [77]	2 [23, 78]

Some studies tested for multiple candidate genes. Therefore, the number of inquiries, as shown here, exceeded the number of association studies. The number of positive or negative associations reflects the inquiry conclusions, based on the *p* values reported. Among the 61 inquiries that matched inclusion criteria, 18 showed positive associations with idiopathic scoliosis. RORA was evaluated in one study [63], but no polymorphisms were found

*One study for IL6 [32] was inconclusive, as the single nucleotide polymorphism verified was not polymorphic in the study cohort

Studies tested for correlations to curve predisposition, progression (severity), and in some cases, comorbidities such as low bone mineral density or abnormal anthropometric features. From the studies listed in Table 1, we highlighted genetic regions associated/linked to IS (Table 2) and provided details of polymorphisms for which no association was detected (Table 3).

**Table 2:** Positive genetic associations with idiopathic scoliosis

Gene	Number of cases/controls	Phenotype Cobb angle	Population [Reference]	Results
**MATN1** 1p35	50/100	>5º	Italian [19]	Microsatellite (short tandem repeat) polymorphism 3′ untranslated region: predisposition (*p* = 0.0242)
	419/750	Not mentioned	Chinese [22]	Promoter polymorphism rs1149048: predisposition and progression **(** ***p*** **=** 0.0034*: OR = 1.34)
**TIMP2** 17q25	570/210	>20º	Chinese [30]	Rs8179090: progression—thoracic curve only (*p* = 0.019; OR = 1.707)
**MMP3** 11q22.3	53/206	25º–125º	Italian Caucasian [31]	rs3025058: predisposition—MMP3 5A/5A (*p* = 0.010, OR = 3.34)
**CALM1** 14q24-q31	67 (40 with thoracic curve)/100	>30º (double curve pattern only)	Chinese [45]	rs12885713: predisposition in double curve pattern (*p* = 0.034); rs5871: thoracic curves only (*p* = 0.0102)
**IL6** 7p21	53/206	25º–125º	Italian Caucasian [31]	rs1800895: predisposition—IL6 G/C (*p* = 0.014, OR = 4.84); IL6 G/G (*p* < 0.001, OR = 10.54)
	198/120	>10º	Korean [52]	IL6-572 G → C: comorbidity –lumbar low BMD density in AIS (*p* = 0.0159)
**VDR** 12q13.11	198/120	>10º	Korean [53]	Bsml: predisposition (*p* = 0.0054); lumbar low BMD in IS (*p* = 0.0046)
**TNFRSF11B** (OPG) 8q24	198/0	>10º	Korean [55]	OPG1181 G → C: comorbidity—lumbar low BMD in IS girls (*p* = 0.0010)
**MTNR1B** 11q21-22	Stage I: 472/304 Stage II: 342/347 (umbilical cord blood controls)	>20º	Chinese [62]	rs4753426: predisposition (*p* = 0.045); meta-analysis on 2 stages of cohorts—(allele *p* = 0.0064, genotype *p* = 0.015)
**TPH1** 11p15.3-p14	103/107	>30º	Chinese [64]	rs10488682: predisposition (allele ***p*** **=** **0.002**, OR = 2.909, genotype ***p*** **=** **0.001**)
**ESR1** (alpha) 6q25.1	202/174	25º–125º	Chinese [68]	rs9340799 (XbaI): predisposition in females with AIS (*p* = 0.010)
	304/0	>10º + rotational prominence	Japanese [67]	rs9340799 (XbaI): progression (*p* = 0.002)
	67 (40 with thoracic curve)/100	>30º (double curve pattern only)	Chinese [45]	rs2234693 (PvuII): predisposition in double curve pattern (*p* = 0.014); curves > 40º (*p* = 0.0128); thoracic curves only (*p* = 0.0184)
**ESR2** (beta) 14q23.2	218/140	12º–135º	Chinese [73]	rs1256120: predisposition (*p* = 0.037, OR = 1.88) and progression (*p* = 0.005)
**GPER** (GPR30)	389/338	>15º	Chinese [74]	Predisposition: rs3808351 (***p*** **=** **0.004**); rs10269151 (***p*** **=** **0.048)**; rs426655s3 (***p*** **=** **0.028**)
**IGF1** 12q23.2	506/227	>20º	Chinese [77]	rs5742612: progression—IGF1 promoter associated with curve severity (*p* = 0.042)

All studies were candidate gene case–control studies except for MATN1 [19], which was a linkage analysis study, and ESR1 [67] and TNFRSF11B (OPG) [55], which were case-only studies. The association with ESR1 found by Esposito et al. [69] was omitted from this table because no statistics were performed in that study

*AIS* adolescent idiopathic scoliosis, *IS* idiopathic scoliosis, *BMD* bone mineral density

* *p* value in bold reflects correction for multiple testing (Bonferroni [64, 74] or 10,000 permutations [22])

**Table 3:** Negative candidate gene studies, no association with idiopathic scoliosis detected

Gene (years)	SNP tested	Number of cases/controls	Phenotype cobb angle	Population
**MATN1** (2011)	rs1149048	798/1,239	>15º	Japanese [23]
**LOX1-5** (2011)	A total of 14 SNPs tested in replication cohort	Discovery cohort: 138/411 Replication cohort: 400/506	>10º	Caucasian–American [24]
**MMP3** (2011)	rs3025058	126/197	64.7º ± 19.2º	Hungarian [33]
**MMP3** (2010)	rs3025058	487/494*	not measured	Chinese [32]
**DPP9** (2008)	rs10406145, rs11670570, rs2286367, rs2277733, rs732631	571/236	>20º	Chinese [37]
**BMP4** (2011)	rs4898820	126/197	64.7º ± 19.2º	Hungarian [33]
**LEP** (2011)	rs7799039	126/197	64.7º ± 19.2º	Hungarian [33]
**IL6** (2011)	rs1800795	126/197	64.7º ± 19.2º	Hungarian [33]
**RANKL** (2009)	rs12721445, rs2277438	198/0	>10º	Korean [55]
**RANK** (2009)	RANK-421 C → T, RANK-575 C → T	198/0	>10º	Korean [55]
**MTNR1A** (2011)	rs6847693, rs2165667, rs2165666	589/1,533	>40º	Caucasian American [60]
**MTNR1A** (2008)	rs2119882	226/277	>10º	Chinese [59]
**MTNR1B** (2011)	10 SNPs tested	589/1,533	>40º	Caucasian–American [60]
**MTNR1B** (2011)	rs4753426	798/1,239	>15º	Japanese [23]
**MTNR1B** (2011)	rs4753426	126/197	64.7º ± 19.2º	Hungarian [33]
**MTNR1B** (2010)	rs4753426	406/479	>10º	American [63]
**MTNR1B** (2006)	rs10830963, rs3781637, rs10830964	473/311*	>20º	Chinese [61]
**TPH1** (2011)	rs1800532, rs10488683, rs211105, rs172423	589/1533	>40º	Caucasian–American [60]
**TPH1** (2011)	rs10488682	798/1239	>15º	Japanese [23]
**ASMT** (HIOMT) (2011)	rs6588807, rs4521942, rs6588810	589/1533	>40º	Caucasian–American [60]
**AANAT** (SNAT) (2010)	rs16968964, rs11077823, rs11077821	406/479	>10º	American [63]
**AANAT** (SNAT) (2008)	tested rs3760138, rs4238989, rs28936679	103/107	>30º	Chinese [64]
**GPR50** (2010)	rs561077, rs13440581	406/479	>10º	American [63]
**PKCD** (2011)	rs1483185, rs3821689, rs17052826, rs13084863	589/1,533	>40º	Caucasian–American [60]
**ESR1** (2010)	rs1256120	798/637	>15º	Japanese [71]
**ESR1** (2006)	rs2234693, rs9340799	540/260	>20º	Chinese [70]
**ESR2** (2010)	rs9340799	798/637	>15º	Japanese [71]
**GHR** (2009)	7 SNPs tested	106/106	>20º	Chinese [78]
**GHR** (2007)	rs6179, rs6176, rs6180, rs6184, exon-3 deletion	510/363*	>20º	Chinese [76]
**IGF1** (2011)	rs5742612	798/1,239	>15º	Japanese [23]
**IGF1** (2009)	rs35767, rs5742612, rs17884626, rs3730195	106/106	>20º	Chinese [78]

Studies that tested associations using microsatellite markers are not listed: Aggrecan, tested in American [18] and Russian [16] cohorts; COL1A1, COL1A2, and COL2A1, tested in 4 Caucasian families with autosomal dominant inheritance [15]; COL1A2, ELN, and FBN1, tested in 11 Caucasian families with autosomal dominant inheritance [14]; and MTNR1A, tested in an American cohort [17]

*SNP* single nucleotide polymorphism

* Female cohort

### Connective tissue structure

Structural proteins are those involved in the extracellular matrix. The genes encoding fibrillin (*FBN1*), elastin (*ELN*), collagen I A1 and A2 (*COL1A1*, *COL1A2*), collagen II A1 (*COL2A1*), and aggrecan (*ACAN*), showed no association with IS on linkage analysis and/or transmission disequilibrium testing [14–16, 18]. Interestingly, using 50 informative Italian trios, Montanaro and colleagues [19] showed that an intragenic microsatellite (short tandem repeat) polymorphism in the 3′ untranslated region of the matrilin 1 gene (*MATN1*) was associated with adolescent IS. Matrilin 1 is a non-collagenous protein, also known as cartilage matrix protein; it is involved in extracellular matrix assembly and is essential for support of the spine [20]. Further to these results, Chen et al. demonstrated a similar association in a Chinese population sample (419 cases/750 controls), using tag single nucleotide polymorphisms (SNPs) from the HapMap database [21, 22]. The association, however, could not be detected in a larger Japanese cohort (789 cases/1,239 controls) even though the study was sufficiently powered [23]. Based on smaller cohorts, the earlier *MATN1* results may likely be false positives rather than differences related to genetic heterogeneity between populations.

Human lysyl oxidases are enzymes involved in the modeling of collagen and elastin. Despite prior experiments in animal models suggesting a link to scoliosis, no association was found for five genes (*LOX*, *LOX1*, *LOX2*, *LOX3*, *LOX4*) when common polymorphisms were verified in an American population (discovery cohort 138 cases/411 controls, replication cohort 400 cases/506 controls) [24].

Extracellular matrix degradation and remodeling are important for normal endochondral ossification. The process is mainly regulated by matrix metalloproteinases (MMPs) and their inhibitors (tissue inhibitors of metalloproteinases, TIMPs) [25, 26]. TIMP2 is the major TIMP expressed during endochondral ossification and has the capacity to inhibit a broad range of MMPs [27, 28]. The *TIMP2* gene is located at 17q25.3, a region previously identified as linked to IS [29]. A polymorphism in the *TIMP2* promoter was indeed associated with thoracic curve severity (*n* = 354), though not with lumbar curve severity (*n* = 216) or curve predisposition, in a cohort of Chinese females (570 cases/210 controls) [30]. *MMP3* was correlated to curve predisposition in a small Italian cohort (53 cases/206 controls) [31], but not in a larger Chinese cohort (487 cases/494 controls) [32]. Nor was *MMP3* directly associated with IS in a Hungarian sample (126 cases/197 controls), although the authors suggested that *MMP3* might modulate curve susceptibility when interacting with bone morphogenetic protein 4 (BMP4) [33].

Dipeptidyl-peptidase 9 (*DPP9*) is a widely expressed gene coding for a protease that functions in cell adhesion, migration, and apoptosis [34]. Based on its location at 19p13.3, a region identified as linked to IS in two linkage studies [35, 36], it was tested as a candidate in Chinese females (571 cases/236 controls). No association was detected [37].

To summarize, of the structural genes tested, *TIMP2* was positively associated with thoracic curve severity in a Chinese cohort. These results need to be replicated using an independent cohort, especially as certain associations such as *MATN1* or *MMP3* were not replicated using larger cohorts. Furthermore, the linkage and transmission disequilibrium studies that showed negative results may have been underpowered to detect common variants possibly associated with IS, so those genes cannot be ruled out in the general population. Nonetheless, the study showing a negative association between the five lysyl oxidase genes and IS had 80 % power to detect an odds ratio of 1.7–2.0, assuming a dominant model of inheritance with no additive or multiplicative effects, a prevalence in the population of 3 %, and a minor allele frequency of 0.10 [24].

### Bone formation and bone metabolism

Other IS candidate genes are related to bone integrity and formation. Bone morphogenetic proteins are polypeptide growth factors that enhance the differentiation of osteoblasts [38]. BMP4 is able to stimulate de novo bone and cartilage formation [39, 40]. Thus *BMP4* was tested in a Hungarian sample (reference SNP rs4898820) (126 cases/197 controls); no association with IS was found [33]. The group also tested a SNP in the leptin gene (*LEP*) (rs7799039) and found that although it was not directly associated with IS, it may interact with the gene for interleukin-6 (*IL6*) to cause IS. However, no correction for multiple testing was performed in this study. The same group also tested the gene encoding calmodulin 1 (*CALM1*), a calcium-dependent regulatory protein that mediates a large number of proteins and plays a key role in the regulation of bone turnover [41]. *CALM1* had been hypothesized to play a role in IS pathogenesis [42–44]. In a small Chinese sample (67 cases/100 controls), polymorphisms in rs12885713 were found to be associated with the predisposition for a double curve [45].

Generalized low bone mass and osteopenia in the axial and peripheral skeleton have been described in IS, along with bone biopsies showing an abnormal histomorphometric profile of bone cell activity [46–51]. However, the precise mechanisms and causes of bone loss in IS have not been identified. To discover genes associated with osteopenia in IS, genes potentially associated with osteoporosis were tested. *IL6* was found to be associated with curve predisposition in a small Italian cohort (53 cases/206 controls) [31], but this finding was not confirmed in a slightly larger Hungarian sample (126 cases/197 controls) [33]. The same marker was not polymorphic in a larger (487 cases/494 controls) cohort of Chinese females [32]. Although not associated with IS predisposition or severity, a different *IL6* variant was found to be associated with low lumbar bone mineral density in Korean females with IS (198 cases/120 controls) [52]. Furthermore, in a case-only study, the vitamin D receptor gene (*VDR*) interestingly was associated with curve predisposition and low lumbar bone mineral density in a sample of Korean females (198 cases/120 controls) [53], whilst not with curve progression in 304 Japanese females with IS [54]. The genes for receptor activator of nuclear factor-κB (*RANK*), now known as tumor necrosis factor receptor superfamily member 11a NFKB activator (*TNFRSF11A*), as well as RANK ligand (*RANKL*) and osteoprotegerin (*OPG*), now known as tumor necrosis factor receptor superfamily member 11B (*TNFRSF11B*), were tested for association with IS severity and/or low bone mineral density in a case-only study of 198 Korean females [55]. The authors found that *OPG* was associated with low lumbar spine bone mineral density, although *RANK* and *RANKL* were not associated with IS.

To summarize, *CALM1*, *IL6*, *LEP*, and *VDR* seemed to be associated with curve predisposition, and *IL6*, *VDR*, and *OPG* with low bone mineral density. These associations need to be verified in larger cohorts. Furthermore, the negative studies described in this section had such small cohorts that we cannot rule out genetic associations for these candidates without further study.

### Melatonin signaling pathway

Genes related to melatonin were considered IS candidates because chickens and rats with little or no circulating melatonin developed spinal curvature, preventable by the readministration of melatonin [56, 57]. However, the lack of any significant differences in melatonin levels between IS patients and controls suggested that IS in humans might be caused by other components of the melatonin signaling pathway [58]. Therefore, the genes encoding melatonin receptors 1A (*MTNR1A*; *Mel*-*1A*-*R*) and 1B (*MTNR1B*; *MT2*; *Mel*-*1B*-*R*) were tested as candidates. Genetic variants of *MTNR1A* were not associated with IS, in a linkage study of 47 American families with autosomal dominant inheritance [17], nor in a larger Chinese female cohort (226 cases/277 controls) [59] or American cohort (589 cases/1,533 controls) [60]. For *MTNR1B*, Qiu XS et al. [61] found no association between IS and three polymorphisms in the coding region, in Chinese females (473 cases/311 controls). The group later used a 2-phase case–control study (phase I 472 cases/304 controls; phase II 342 cases/347 umbilical cord blood samples as controls) to demonstrate an association between promoter polymorphism rs4753426 and curve predisposition, among Chinese females [62]. In an American population, there were no polymorphisms in the coding region of *MTNR1B*; hence the negative results from the first Chinese study could not be replicated. The rs4753426 promoter polymorphism was tested but did not replicate the association with curve predisposition (406 cases/479 controls) [63]. Associations with the promoter SNP were further ruled out in an independent American study (589 cases/1,533 controls) [60], in Hungary (126 cases/197 controls) [33], and in Japan (798 cases/1,239 controls) [23].

The gene for tryptophan hydroxylase 1 (*TPH1*), an enzyme essential for serotonin biosynthesis (a precursor of melatonin), was associated with curve predisposition in a Chinese cohort (103 cases/107 controls) [64], but not in Japanese (798 cases/1,239 controls) [23] or Caucasian American (589 cases/1,533 controls) cohorts [60]. Other components of the melatonin pathway not associated with IS included aralkylamine *N*-acetyltransferase (*AANAT*), previously known as serotonin *N*-acetyltransferase (*SNAT*), in a Chinese study (103 cases/107 controls) [64] and in the United States (589 cases/1,533 controls) [60]; G protein-coupled receptor 50 (*GPR50*) in the United States (406 cases/479 controls) [63]; and acetylserotonin *O*-methyltransferase (*ASMT*), previously known as hydroxyindole *O*-methyltransferase (*HIOMT*) and protein kinase C delta (*PKCD*) in a separate American cohort (589 cases/1,533 controls) [60]. Although the retinoic acid receptor-related orphan receptor alpha gene (*RORA*) was tested in the United States along with *GPR50*, no polymorphisms were found [63].

To summarize, none of the melatonin pathway-associated genes seemed to be associated with IS. Although an association with *MTNR1A*, *MTNR1B*, and *TPH1* was suggested by smaller studies, larger cohorts did not support their conclusions. These later studies were sufficiently powered to detect any potential effects had they been present.

### Puberty and growth

Because curve pathogenesis in scoliosis coincides with growth and adolescence, genes involved in the somatotrophic and androgenic axes were considered potential IS candidates. The gene encoding cytochrome P450 17α-hydroxylase (*CYP17*) was considered a likely candidate for IS progression because of its critical roles in androgen synthesis. In a cohort of 304 Japanese females, no association with IS was found [54]. Of note, both forms of the estrogen receptor, ESR1 (also known as ERα) and ESR2 (also known as ERβ), are present in osteoblasts and osteoclasts [65], indicating that estrogen regulates osteoblast function directly [66]. The *ESR1* gene has been extensively examined as it contains the polymorphic sites PvuII (rs2234693) and XbaI (rs9340799). XbaI (but not PvuII) was identified as a factor in IS progression in a case-only study of 304 Japanese females [67]; in Chinese females (202 cases/174 controls), it was associated with curve predisposition, progression, and abnormal growth [68]. In a separate Chinese study, analysis of a small cohort of patients with double-curve patterns only (67 cases/100 controls) suggested that PvuII (but not Xba1) was associated with curvature [45]. Using restriction site analysis, Esposito et al. [69] reported that XbaI was associated with low levels of steroids in several Italian females with IS (4 out of 174 cases/104 controls), although no statistical analyses were effected. However, associations with Xba1 and PvuII were not confirmed in a larger cohort of Chinese females (540 cases/260 controls) [70], nor was the association with Xba1 replicated in a larger Japanese study (798 case/637 control) [71]. Although ESR1 is the major estrogen receptor in bone, it has been shown that in females, the *ESR2* gene can modulate the action of ESR1 [72]. In China, the *ESR2* polymorphism rs1256120 was associated with curve predisposition and progression (218 case/140 control) [73], though the association was not confirmed in a larger Japanese cohort (798 cases/637 controls) [71]. Recently, the gene for the novel G protein-coupled estrogen receptor GPER (also known as GPR30) was found to be associated with curve severity, but not curve predisposition, in a sample of Han Chinese (389 cases/338 controls) [74].

There is evidence that estrogen enhances the growth hormone/insulin-like growth factor (IGF-1) axis, in both males and females, and is the main mediator of the accelerated linear growth and increases in bone dimensions observed during early-to-mid puberty [75]. Because accelerated linear growth is related to curve progression, genes that define this process are candidates for inclusion in IS research. Although no association was found between IS and the gene for the growth hormone receptor (*GHR*) in a cohort of Chinese females (510 cases/363 controls) [76], *IGF1* was demonstrably associated with curve severity in a separate cohort of Chinese females (506 cases/227 controls) [77]. Neither gene, however, appeared to be related to IS in a small independent Chinese cohort (106 cases/106 controls) [78]. The lack of association between IS and *IGF1* was further confirmed in a large Japanese cohort (798 cases/1,239 controls) [23].

To summarize, associations in small populations between common polymorphisms of the genes encoding the α- and β-estrogen receptors and IS were not confirmed in two larger studies. Another two studies found no association for *GHR*. Whether *IGF1* or *GPER* are associated with IS needs to be confirmed in larger cohorts.

## Important considerations for candidate gene studies in IS

A successful candidate gene is one that demonstrates a truly significant association with a disease. The truth of an association is suggested by the power of the study and proven by replication studies. Generally, the success rate for candidate gene studies has been poor. A 2002 review of 603 published association studies for human disease showed replication of results in only 1 % of the studies [79]. This lack of success is indicative of poor study design in defining the phenotype to be tested, the selection of controls, selection of genetic markers, and adequate sample size [80, 81].

For case–control studies, whether they are candidate gene or genomic association studies, the case population has to be well defined to avoid genetic and environmental heterogeneity that would decrease the power of detection and hinder replication results. This is of particular concern in IS because curve phenotype ostensibly derives from various underlying etiologies. Some studies have attempted to refine the phenotype with clinical parameters, such as considering only double curves [45] or pronounced curves (greater than 40º) [16, 33, 60]. The recent identification of biochemical endophenotypes for IS [82–84] may thus potentially reduce the heterogeneity confounding current genetic studies. Endophenotypes as conceived by Gottesman and colleagues [85, 86] are heritable, quantitative traits associated with an illness both epidemiologically and conceptually, in the sense of being on the putative path from genes to molecular biological mechanisms. They are state-independent (i.e., present not only during acute illness), co-segregate within families, and may appear in unaffected relatives of individuals with the disorder because they represent vulnerability for the disorder, but are at a higher prevalence in affected individuals as compared with the general population. For complex phenotypes such as IS, an optimal case definition will contain both clinical and biologically relevant information, and this definition will likely change over time as more information becomes available [80].

**Fig. 2:**
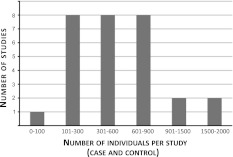
Total number of individuals per candidate gene study A larger cohort will increase the power of a study to detect associations, particularly if the trait is characterized by genetic heterogeneity. Most candidate gene studies for idiopathic scoliosis fell short of the 1,000+ individuals required to demonstrate association with a risk allele of low-to-moderate effect

It is possible that locus or allelic heterogeneity contributes to observed variation in IS. Therefore, detection of an association would require a larger sample size, regardless of disease prevalence [87]. Most candidate gene studies for IS had small cohorts, so it is difficult to discern whether associations were not replicated in other cohorts (same or different ethnic background) as a consequence of genetic heterogeneity or ascertainment bias (e.g., “winner’s curse”) [88] (Fig. 2). Based on estimations from Hattersley and McCarthy [89], a study needs thousands of individuals to detect a common variant of a risk allele with a low-to-moderate effect. For example, for an allele with a frequency of 20 % in controls, detection of a susceptibility allele at a 0.01 level of significance with 90 % power would require 1,255 individuals (assuming an odds ratio of 1:3). Among the 34 candidate gene studies reviewed here, only three recent association studies had more than 1,000 participants [23, 60, 71], all three showing negative results. Furthermore, because it is well known that originally reported effect sizes are likely to be biased upward [88, 90, 91], a replication study should calculate its estimated sample size based on the anticipation of an effect smaller in size than the one originally reported.

## Genome-wide studies

We examined 14 genome-wide family-based linkage studies for IS and two GWAS, all published after the year 2000. All defined the phenotype as a lateral curvature of the spine with a minimum Cobb angle of 10º.

Typically, two approaches are used for linkage analysis: parametric and non-parametric. The parametric logarithm of odds (LOD) score method is a model-dependent approach. Mode of inheritance, crossover rate, morbid gene frequency, trait penetrance, phenocopy rate, and allele frequencies have to be provided. Either single-point or multipoint calculations can be made. In single-point analysis, linkage between a trait and a given marker/locus is indicated by an LOD score ≥3 for autosomes (odds ratio 1,000:1), or ≥2 for the X chromosome (odds ratio 100:1). Conversely, a LOD score ≤−2 is evidence for exclusion of the locus (odds less than 1:100 that the locus is linked). For multipoint analysis, an LOD score of 3.3 (*p* value around 10^−5^) is considered significant linkage [92]. The second approach to linkage analysis, a non-parametric or model-independent approach, is based on the hypothesis that relatives sharing the same trait should share heritable alleles. Other than allele frequencies, the other criteria necessary for parametric analysis are not useful. A LOD score of 2 (*p* value of 7 × 10^−4^) is suggestive of linkage, a LOD score of 3.3 (*p* value of 2 × 10^−5^) is considered significant linkage, and a LOD score of 5.4 (*p* value of 3 × 10^−7^) is considered highly significant linkage. Importantly, significant observations should be reproducible in an independent cohort, with *p* < 0.01, according to Lander and Kruglyak [92].

### Parametric linkage analysis

**Fig. 3:**
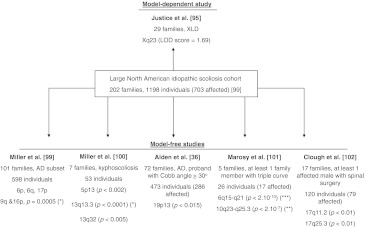
Six genome-wide linkage studies derived from a large idiopathic scoliosis cohort. One model-dependent [95] and five model-free studies [36, 99–102] are shown, with their major findings. Levels of significance are indicated, based on recommendations by Lander E and Kruglyak L [92]: (*asterisk*), suggestive; (*double* *asterisk*), significant; (*triple* *asterisk*), highly significant. *AD* autosomal dominant, *XLD* X-linked dominant, *LOD* logarithm of odds

With linkage studies, the number of meioses is more pertinent than the number of families, as different susceptibility genes may segregate among different families [93]. For this reason, a study with one or several large families containing many affected members is optimal for detection of susceptibility loci. In the present review, seven studies using informative families identified nine loci linked to IS (Table 4) [29, 35, 94–98]. In four of these studies, single multiplex informative families displayed autosomal dominant inheritance reaching significance, according to the LOD threshold required for valid linkage [29, 94, 96, 98]. A fifth one showed X-linked dominant transmission among 29 families (202 individuals identified, overall LOD score = 1.69, of which a single family contributed a LOD score = 2.23) (Fig. 3; Table 4) [95]. With regards to locus 18q12.1-12.2, it is important to note that the LOD score was below 3 when individuals with pectus excavatum were removed from the analysis (LOD_max AIS_ = 2.77) [96], suggesting that in this family, both phenotypes were linked to the same locus but that the loss of power was likely due to reduced sample size.

**Table 4:** Genome-wide parametric linkage results for idiopathic scoliosis

Reference	Pedigree characteristics	Cobb angle	Genetic inheritance	Locus	Statistics
Single-family studies					
Salehi et al. [94]	4 generations, 11 affected out of 17	10º–20º	AD, pen = 1	17p11	*Z*_max_ = 3.20
Justice et al. [95]	6 affected	>10º	XLD, pen F = 0.90, pen M = 0.79	Xq22.3-q27.2	LOD = 2.23
Ocaka et al. [29]	5 generations, 8 affected out of 21	15º–65º	AD, pen = 0.80	9q31.2-q34.2	*Z*_max_ = 3.64
Gurnett et al. [96]	5 generations, 9 affected out of 22 + 4 pectus excavatum	15º–70º	AD, pen = 0.80	18q12.1-q12.2	LOD_max AIS+PE_ = 3.86 LOD_max AIS_ = 2.77
Edery et al. [98]	3 generations, 11 affected out of 18	15º–41º	AD, pen not noted	3q12.1 5q13.3	*Z*_max_ = 3.00 *Z*_max_ = 3.01
Multi-family studies					
Chan et al. [35]	7 families, 25 affected	>10º	AD, pen = 0.80	19p13.3	LOD = 4.48
Ocaka et al. [29]	2 families, 16 affected out of 49	11º–55º	AD, pen = 0.80	17q25.3-qtel	LODcomb = 3.78
Raggio et al. [97]	7 families, 18 affected out of 50	>10º	AR, α = 1 AD, α = 1	12p	HLOD = 3.2 HLOD = 3.7

*AD* autosomal dominant, *AR* autosomal recessive, *F* female, *M* male, *pen* penetrance, *XLD* X-linked dominant

In addition to the six primary regions noted above, three secondary loci were identified using cohorts of multiple smaller families [29, 35, 97]. For the most part, these families included less than 6 affected individuals each, except for family SC36, which had 10 affected individuals out of 25 [29]. The combined LOD score reported corresponded to the sum of genetic contributions from each family, as none of the pedigrees reached statistical significance independently. According to the published data, family SC36 was the most genetically informative, as it showed a LOD score of 2.64 at 17q25.3 [29].

To summarize, nine loci were identified as linked to IS. Of these, seven met the threshold for significance: 3q12.1, 5q13.3, 9q31.2-34.2, 12p, 17p11, 19p13.3, and Xq22.3-27.2.

### Non-parametric linkage analysis

Six of the genome-wide linkage studies were subsets of a larger North American cohort of 202 families (1,198 individuals, including 703 individuals with IS). Five of these studies were model-independent [36, 99–102], while one was a parametric study described in the previous section [95] (Fig. 3). Various traits were analyzed (kyphoscoliosis, scoliosis, gender-related severe scoliosis, curve pattern), illustrating the difficulty in defining the IS phenotype. Regions on chromosomes 6p, 6q, 9q, 16p, and 17p were found to be linked to curve susceptibility in 101 families with autosomal dominant inheritance [99]. Three other loci (5p13, 13q13.3 and 13q32) were linked to kyphoscoliosis (defined by a sagittal curve >40º) in a subgroup of seven families [100]. One locus (19p13) was linked to curve progression in families whose probands had a Cobb angle ≥30º [36]. Two loci were linked to triple curve scoliosis (6q15-q21 and 10q23-q25.3) [101], and a chromosomal region (17q) to male-specific severe scoliosis [102]. Not all these regions were deemed significant by Lander and Kruglyak standards, however [92] (Fig. 3).

Using different cohorts, Chan et al. [35] identified the 19p13.3 locus as linked to IS in Chinese families and Gao et al. [103] suggested linkage between the gene for the calcium-dependent adhesion transmembrane protein cadherin 7 type 2 (*CDH7*) (8q12) and IS in American individuals of European descent (Table 5).

**Table 5:** Genome-wide nonparametric linkage results for idiopathic scoliosis

Study	Pedigree characteristics	Cobb angle	Locus	Statistics
Chan et al. [35]	7 families 25 affected	> 20º	19p13.3	NPL = 5.36, *p* = 3.10^−5^ (**)
Gao et al. [103]	53 families 130 affected	15º–113º	8q12 (*CDH7*)	Zlr = 2.72, *p* = 2.10^−4^ (*) *p*_TDTae_ = 2.10^−4^ *p*_HHRR_ = 0.001

*NPL* nonparametric linkage, model-independent

Significant (**) and suggestive (*) results, as per recommendations by Lander E and Kruglyak L [92]

Thus, of the seven model-independent studies [35, 36, 99–103], only three loci reached statistical significance: 6q15-q21, 10q23-q25.3 and 19p13.3.

### Important candidate regions in genome-wide linkage studies

Taken together, the parametric and nonparametric genome-wide linkage studies revealed various regions of interest to IS. In 2005, Miller et al. [99] first identified 9q31-q34 with suggestive linkage (*p* < 0.006), a finding supported in 2007 by Ocaka et al. [29] in a single family (*Z*_max_ = 3.64). According to Lander and Kruglyak [92], a *p* value of 0.01 signifies confirmation of linkage in a replication cohort. Also, although not the strongest locus according to the standards discussed here, the region at 17q25 was noteworthy. First identified by Ocaka et al. [29] in 2007 using two families (*Z*_max_ = 2.64 and *Z*_max_ = 1.81), this region was also identified by Clough et al. [102] in 2010 (*p* < 0.01). Of the 19 families described, 18 had at least one individual with spinal surgery or bracing, suggesting that this locus was linked to curve severity.

### Genome-wide association studies

Two GWAS on IS were recently published [104, 105]. One consisted of a discovery cohort of 419 families (total of 1,122 individuals) and three replication cohorts, in which 327,000 SNPs were genotyped [104]. While statistical thresholds were not clearly mentioned, transmission disequilibrium testing on the discovery cohort followed by case–control comparisons on the replication cohorts identified SNPs located on chromosome 3 in the region of the L1 cell adhesion molecule gene (*CHL1*)/LOC642891 [rs10510181 OR = 1.37, CI = (1.20–1.58), *p* = 8.22.10^−7^]. Furthermore, the authors reported the replicated association of the Down syndrome cell adhesion molecule gene (*DSCAM*) [combined results for rs2222973 OR = 0.59, CI = (0.48–0.74), *p* = 1.46.10^−6^]. *Dscam* partial knockdowns had previously been shown to produce crooked tails in zebrafish embryos [106]. These two genes are involved in axon guidance pathways, evidence of a potential neuropathology underlying IS. Modest associations were found in clusters within the 9q31-q34 locus, but the authors failed to replicate previous observations concerning linkage/association of *CDH7*.

The second study consisted of a discovery cohort composed of 1,050 Japanese cases and 1,474 Japanese controls and a replication cohort of 326 affected adolescents, and 9,823 controls, for which, here again, 327,000 SNPs were genotyped [105]. Three SNPs (rs11190870, rs625039 and rs11598564) reached genome-wide significance (*p* value of 1 × 10^−7^) and were located near the ladybird homeobox 1 (*LBX1*) gene locus (10q24.32). Even hypothesizing an abnormal somatosensory etiology for IS in which *LBX1* could be involved, the role of this gene has to be further explored. As none of the previous GWAS results were found, ethnic and/or genetic heterogeneity may be assumed.

## Conclusions

In this comprehensive review of the genetics underlying IS, we analyzed 50 studies. Findings involved genes related to connective tissue structure, bone formation/metabolism, melatonin signaling pathways, puberty and growth, and axon guidance pathways. The genetic basis for the etiology and prognosis of IS remains elusive, however. As with other genetic studies, the goals were to identify susceptibility genes for IS, define disease modifying genes, and explain why some curves progress to severity while others do not (genes that could be shared with the asymptomatic healthy population). The major difficulty faced by IS genetic studies is phenotypic and genetic heterogeneity. We found that IS genetic studies were overrepresented by underpowered studies that suggested an association, and then by underpowered replication studies that could not confirm or refute the original hypotheses. Although an increase in the number of individuals generally enhances the power of a study to detect an effect, genetic heterogeneity in complex diseases like IS is a major obstacle that cannot be overcome by such means alone.

With the advent of high-throughput technologies, future studies will be able to genotype a greater number of markers to possibly identify causal variants. However, understanding the difficulties surrounding this complex phenotype and the strengths and weaknesses of prior studies is crucial for progress in defining the genetics of this deformity. The use of biological endophenotypes such as those defined by Moreau et al. as well as restricted clinical definitions may facilitate the partitioning of variation and increase the power of detecting genetic associations. In addition, replication studies should use power analysis to minimize the possibility of false negatives. Further, when multiple polymorphisms are tested, an appropriate correction for significance thresholds needs to be applied.
